# Mitochondrial-ER Contact Sites and Tethers Influence the Biosynthesis and Function of Coenzyme Q

**DOI:** 10.1177/25152564251316350

**Published:** 2025-02-03

**Authors:** Noelle Alexa Novales, Hadar Meyer, Yeynit Asraf, Maya Schuldiner, Catherine F. Clarke

**Affiliations:** 1 Department of Chemistry & Biochemistry, Molecular Biology Institute, 8784 University of California, Los Angeles, CA, USA; 2 Department of Molecular Genetics, 34976 Weizmann Institute of Science, Rehovot, Israel

**Keywords:** coenzyme Q, mitochondria, ER-mitochondrial encounter structure, artificial tether

## Abstract

Coenzyme Q (CoQ) is an essential redox-active lipid that plays a major role in the electron transport chain, driving mitochondrial ATP synthesis. In *Saccharomyces cerevisiae* (yeast), CoQ biosynthesis occurs exclusively in the mitochondrial matrix via a large protein-lipid complex, the CoQ synthome, comprised of CoQ itself, late-stage CoQ-intermediates, and the polypeptides Coq3-Coq9 and Coq11. Coq11 is suggested to act as a negative modulator of CoQ synthome assembly and CoQ synthesis, as its deletion enhances Coq polypeptide content, produces an enlarged CoQ synthome, and restores respiration in mutants lacking the CoQ chaperone polypeptide, Coq10. The CoQ synthome resides in specific niches within the inner mitochondrial membrane, termed CoQ domains, that are often located adjacent to the endoplasmic reticulum-mitochondria encounter structure (ERMES). Loss of ERMES destabilizes the CoQ synthome and renders CoQ biosynthesis less efficient. Here we show that deletion of *COQ11* suppresses the respiratory deficient phenotype of select *ERMES* mutants, results in repair and reorganization of the CoQ synthome, and enhances mitochondrial CoQ domains. Given that ER-mitochondrial contact sites coordinate CoQ biosynthesis, we used a Split-MAM (Mitochondrial Associated Membrane) artificial tether consisting of an ER-mitochondrial contact site reporter, to evaluate the effects of artificial membrane tethers on CoQ biosynthesis in both wild-type and *ERMES* mutant yeast strains. Overall, this work identifies the deletion of *COQ11* as a novel suppressor of phenotypes associated with *ERMES* deletion mutants and indicates that ER-mitochondria tethers influence CoQ content and turnover, highlighting the role of membrane contact sites in regulating mitochondrial respiratory homeostasis.

## Introduction

Coenzyme Q (ubiquinone or CoQ) is an essential redox-active lipid molecule found in the plasma membranes and endomembranes of all eukaryotes (Banerjee et al., 2022; Guerra and Pagliarini, 2023). Its canonical function in the mitochondrial electron transport chain involves carrying electrons and protons between respiratory complexes to establish a membrane potential and drive ATP synthesis. CoQ also supports pyrimidine biosynthesis, proline and sulfide catabolism, fatty acid beta-oxidation, and choline degradation (Banerjee et al., 2022; Guerra and Pagliarini, 2023). Additionally, the fully reduced CoQH_2_ (ubiquinol) acts as a lipid-soluble antioxidant capable of ameliorating peroxidation of lipids in cellular membranes and lipoproteins (Frei et al., 1990; Wang et al., 2024).

Membrane localization of CoQ is dependent on its hydrophobic tail, which is comprised of a species-specific number of isoprene units (denoted by *n* in CoQ_n_) (Okada et al., 1996). This polyisoprenyl tail anchors CoQ in the mid-plane of lipid bilayers, while its fully substituted benzoquinone head group affords redox activity, permitting electron and proton transfer in diverse biological pathways (Frei et al., 1990; Banerjee et al., 2022; Guerra and Pagliarini, 2023; Wang et al., 2024).

Patients with diminished CoQ levels can be treated with CoQ_10_ supplements (Garrido-Maraver et al., 2014), though its uptake is limited because the hydrophobicity of CoQ_n_ with n = 6–10 hinders aqueous transport and delivery to the inner mitochondrial membrane (Fernandez-Del-Rio et al., 2020; Guile et al., 2023). Thus, an understanding of regulatory elements directly associated with the CoQ biosynthetic machinery and putative transport mechanisms is critical for elucidating its endogenous biosynthesis and distribution.

**Figure 1.**
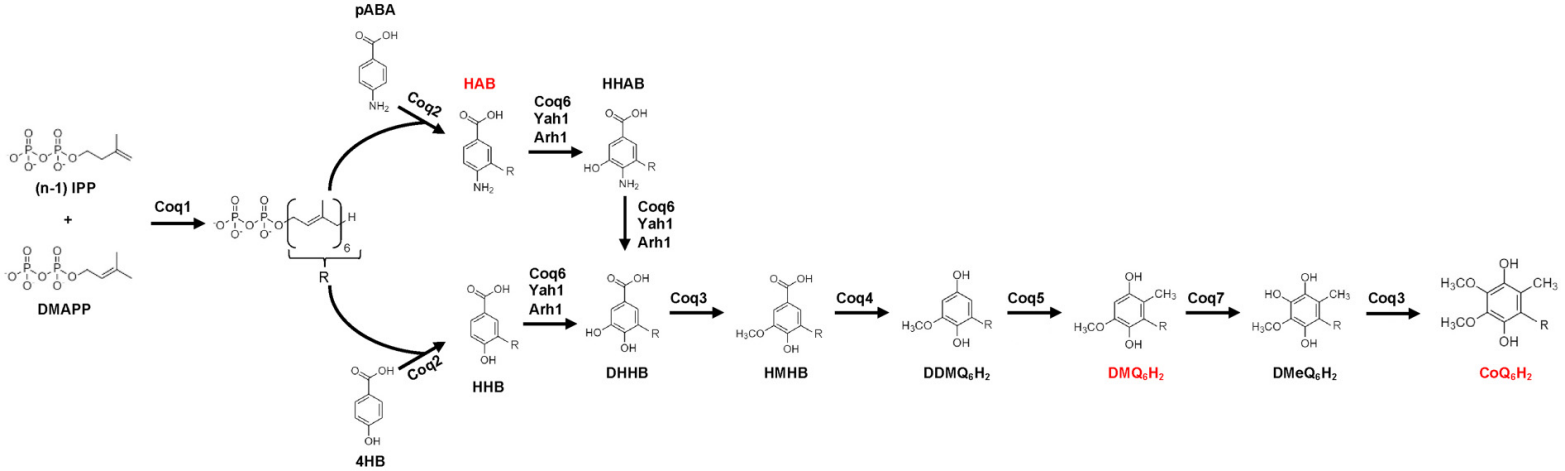
The CoQ biosynthetic pathway. Representation of the CoQ_6_ biosynthetic pathway in yeast. Intermediates analyzed are indicated in red text. Abbreviations: IPP, isopentenyl pyrophosphate; DMAPP, dimethylallyl pyrophosphate; 4HB, 4-hydroxybenzoic acid; pABA, 4-aminobenzoic acid; HHB, 3-hexaprenyl-4-hydroxybenzoic acid; HAB, 3-hexaprenyl-4-aminobenzoic acid; HHAB, 3-hexaprenyl-4-amino-5-hydroxybenzoic acid; DHHB, 4,5-dihydroxy-3-hexaprenylbenzoic acid; HMHB, 4-hydroxy-5-methoxy-3-hexaprenylbenzoic acid; DDMQ_6_H_2_; 2-methoxy-6-hexaprenyl-1,4-benzohydroquinone; DMQ_6_H_2_, 2-methoxy-5-methyl-6-hexaprenyl-1,4-benzohyrdroquinone; DMeQ_6_H_2_, 3-methyl-6-methoxy-2-hexaprenyl-1,4,5-benzenetriol.

CoQ biosynthesis involves several pathways that converge within the mitochondria to generate the essential lipid molecule (Guerra and Pagliarini, 2023). Namely, the components that generate the polyisoprenyl tail moiety are derived from the mevalonate pathway, while the head group, 4-hydroxybenzoic acid (4HB), is derived predominantly from tyrosine in eukaryotes. Yeast can also use para-aminobenzoic acid (pABA), derived from chorismate, as an alternative head group precursor (Figure 1) (Marbois et al., 2010; Pierrel et al., 2010). In yeast, prenylation of pABA and 4HB by the Coq2 polypeptide yields the early intermediates, 3-hexaprenyl-4-aminobenzoic acid (HAB) and 3-hexaprenyl-4- hydroxybenzoic acid (HHB), respectively (Figure 1).

In *Saccharomyces cerevisiae*, biosynthesis of CoQ_6_ requires fourteen nuclear-encoded mitochondrial proteins (Coq1-Coq11, Yah1, Arh1, and Hfd1) (Figure 1) (Awad et al., 2018; Guerra and Pagliarini, 2023; Pelosi et al., 2024). The polypeptides Coq3-Coq9 and Coq11 form a megadalton (MDa) complex, the CoQ synthome; correct assembly of this metabolon is required for efficient CoQ_6_ biosynthesis (Hsieh et al., 2007). The CoQ synthome can be visualized as discrete puncta within mitochondria, termed CoQ domains, using select Coq polypeptides tagged with a fluorescent marker at their endogenous loci (Eisenberg-Bord et al., 2019; Subramanian et al., 2019). Individual deletion of the *COQ1*-*COQ9* genes halts CoQ_6_ biosynthesis and respiratory growth on nonfermentable medium (Awad et al., 2018). These *COQ* gene deletions also disrupt CoQ domains and disperse Coq polypeptide fluorescence throughout mitochondria (Subramanian et al., 2019). This is due to the loss of the Coq polypeptide components of the CoQ synthome (Awad et al., 2018), and to loss of late-stage polyprenylated CoQ-intermediates (Subramanian et al., 2019), which are also required for CoQ synthome formation and complex integrity (Tran and Clarke, 2007; Wang and Hekimi, 2019).

CoQ synthome assembly and efficient CoQ biosynthesis also depend on the endoplasmic reticulum-mitochondria encounter structure (ERMES) (Eisenberg-Bord et al., 2019; Subramanian et al., 2019). ERMES is comprised of four proteins (ER protein Mmm1, mitochondrial outer membrane proteins Mdm10 and Mdm34, and cytosolic linker protein Mdm12) that tether ER to mitochondria and facilitate phospholipid transport (Kornmann et al., 2009). Interestingly, both ERMES complexes and CoQ domains colocalize into discrete puncta (Eisenberg-Bord et al., 2019). Deletion of any of the ERMES subunits results in a destabilized CoQ synthome (Eisenberg-Bord et al., 2019) and decreased number of CoQ domains (Subramanian et al., 2019). Additionally, *ERMES* mutants accumulate early CoQ_6_-intermediates, a hallmark of inefficient CoQ_6_ biosynthesis (Eisenberg-Bord et al., 2019). Together, these data demonstrate CoQ synthome assembly and efficient CoQ_6_ biosynthesis depend on ERMES complex formation.

Membrane contact sites constitute essential signaling platforms that mediate interorganelle transport processes. In yeast, ERMES contact sites enable phospholipid transport between the ER and mitochondria (Kornmann et al., 2009; Tan et al., 2013). Three ERMES subunits (Mmm1, Mdm12, and Mdm34) possess synaptotagmin-like mitochondrial lipid binding protein (SMP) domains that form a hydrophobic tunnel poised for lipid transport (Kornmann et al., 2009; Tan et al., 2013; AhYoung et al., 2015; Wozny et al., 2023). Given the colocalization of ERMES puncta with CoQ domains, and the structural and biochemical evidence for lipid trafficking via ERMES, the ERMES complex is an attractive candidate for modulating intracellular CoQ distribution (Guile et al., 2023). However, its ability to bind or transport CoQ has not been evaluated.

*MDM12*, which encodes the cytosolic ERMES subunit, is co-expressed with the *COQ10* gene via a bidirectional promoter, suggesting a putative functional relationship or physical interaction between their gene products (Hibbs et al., 2007; Okamura et al., 2015). *COQ10* encodes a highly conserved CoQ chaperone polypeptide, necessary for respiratory electron transport (Barros et al., 2005; Allan et al., 2013; Tsui et al., 2019). Deletion of *COQ10* disrupts spatial coordination between the CoQ domains and ERMES puncta, as the percent of complex colocalization is significantly reduced in the *coq10*Δ mutant (Eisenberg-Bord et al., 2019). Although deletion of *MDM12* does not significantly impact Coq10 polypeptide levels (Eisenberg-Bord et al., 2019), the *coq10*Δ mutant has severely attenuated Mdm12 polypeptide content due to transcriptional/translational interference from the expression cassette used to replace the *COQ10* open reading frame (Novales et al., 2024). The depletion of Mdm12 in the *coq10*Δ mutant results in the inability to form the ERMES complex (Kornmann et al., 2009). Consequently, a subset of the phenotypes previously attributed to the *coq10*Δ mutant (Bradley et al., 2020), including destabilization of the CoQ synthome and inefficient CoQ biosynthesis instead result from ERMES dysfunction (Novales et al., 2024).

Intriguingly, deletion of the *COQ11* gene rescues defects associated with the *coq10*Δ mutant yeast, including those caused by ERMES disruption (Bradley et al., 2020; Novales et al., 2024). *COQ11* encodes a short chain dehydrogenase reductase (SDR) of unknown function that interacts with the CoQ synthome (Allan et al., 2015). The *coq11*Δ mutant possesses an enlarged CoQ synthome, as demonstrated by two-dimensional Blue Native/SDS-PAGE and fluorescence microscopy (Subramanian et al., 2019; Bradley et al., 2020), suggesting the Coq11 polypeptide may negatively modulate CoQ synthome assembly. Given that *COQ11* deletion is able to rescue *coq10*Δ phenotypes irrespective of ERMES integrity, we investigated whether *COQ11* deletion could remedy phenotypes associated with *ERMES* mutants. We also sought to evaluate whether expression of artificial molecular tethers that mediate ER-mitochondrial contacts affect CoQ biosynthesis. Here we show that the respiratory growth of select *ERMES* deletion mutants can be mitigated by deletion of *COQ11*, highlighting the possibility of unique functions for individual ERMES subunits. Overall, we identify the deletion of *COQ11* as a novel suppressor of *ERMES* mutant phenotypes, suggesting a potential broader role for Coq11 as a modulator of mitochondrial function and mitochondrial-ER CoQ trafficking. Furthermore, our results suggest that ER-mitochondrial tethers may influence the content and turnover of CoQ, highlighting the role of membrane contact sites in regulating mitochondrial respiratory homeostasis.

## Results

### Deletion of *COQ11* in Select *ERMES* Mutants

Given deletion of *COQ11* alleviates the ERMES-related phenotypes manifested by the *coq10*Δ mutant (Novales et al., 2024), we questioned if phenotypes associated with *ERMES* mutants can also be mitigated by the subsequent deletion of *COQ11*. The *COQ11* open reading frame was successfully deleted in the *mmm1*Δ, *mdm10*Δ, and *mdm34*Δ mutants (Table 1).

**Table 1.** Yeast Strains Used in This Study.

Strain	Genotype	Source
W303 1B	*MAT α, ade2-1 can1-100 his3-11,15 leu2-3,112 trp1-1 ura3-1*	R. Rothstein^a^
BY4741	*MAT a his3Δ1 leu2Δ0 met15Δ0 ura3Δ0*	(Brachmann et al., 1998)
JM6	*MAT a his-4 ρ^0^*	J. E. McEwen^b^
JM8	*MAT α ade-1 ρ^0^*	J. E. McEwen^b^
W303a *coq2*Δ	*MAT a, ade2-1 can1-100 his3-11,15 leu2-3,112 trp1-1 ura3-1 coq2::HIS3*	(Ashby et al., 1992)
W303 1B *coq3*Δ	*MAT α, ade2-1 can1-100 his3-11,15 leu2-3,112 trp1-1 ura3-1 coq3::LEU2*	(Do et al., 1996)
W303a *coq4*Δ	*MAT a, ade2-1 can1-100 his3-11,15 leu2-3,112 trp1-1 ura3-1 coq4::TRP1*	(Hsu et al., 2000)
W303 1B *coq5*Δ	*MAT α, ade2-1 can1-100 his3-11,15 leu2-3,112 trp1-1 ura3-1 coq5::HIS3*	(Barkovich et al., 1997)
W303a *coq6*Δ	*MAT a, ade2-1 can1-100 his3-11,15 leu2-3,112 trp1-1 ura3-1 coq6::LEU2*	(Gin et al., 2003)
W303 1B *coq7*Δ	*MAT α, ade2-1 can1-100 his3-11,15 leu2-3,112 trp1-1 ura3-1 coq7::LEU2*	(Marbois and Clarke, 1996)
W303a *coq8*Δ	*MAT a, ade2-1 can1-100 his3-11,15 leu2-3,112 trp1-1 ura3-1 coq8::HIS3*	(Hsu et al., 2000)
W303 1B *coq9*Δ	*MAT α, ade2-1 can1-100 his3-11,15 leu2-3,112 trp1-1 ura3-1 coq9::URA3*	(Johnson et al., 2005)
W303a *coq10*Δ	*MAT a, ade2-1 can1-100 his3-11,15 leu2-3,112 trp1-1 ura3-1 coq10::HIS3*	(Barros et al., 2005)
W303 1B *coq11*Δ	*MAT α ade2-1 his3-1,15 leu2-3,112trp1-1 ura3-1 coq11::LEU2*	(Bradley et al., 2020)
W303a *mmm1*Δ	*MAT a, leu2-3,-112; his3-11,-15; trp1-1; ura3-1; ade2-1; can1-100 mmm1::KanMX*	(Tan et al., 2013)
W303a *mmm1*Δ*coq11*Δ	*MAT a, leu2-3,-112; his3-11,-15; trp1-1; ura3-1; ade2-1; can1-100 mmm1::KanMX coq11::LEU2*	This work
W303a *mdm10*Δ	*MAT a, leu2-3,-112; his3-11,-15; trp1-1; ura3-1; ade2-1; can1-100 mdm10::HIS3*	(Tan et al., 2013)
W303a *mdm12*Δ	*MAT a, leu2-3,-112; his3-11,-15; trp1-1; ura3-1; ade2-1; can1-100 mdm12::HIS3*	(Tan et al., 2013)
W303a *mdm34*Δ	*MAT a, leu2-3,-112; his3-11,-15; trp1-1; ura3-1; ade2-1; can1-100 mdm34::KanMX*	(Tan et al., 2013)
W303a *mdm34*Δ*coq11*Δ	*MAT a, leu2-3,-112; his3-11,-15; trp1-1; ura3-1; ade2-1; can1-100 mdm34::KanMX coq11::LEU2*	This work
W303a Coq9-yeGFP Aco2-mCherry	*MAT α, ade2-1 can1-100 his3-11,15 leu2-3,112 trp1-1 ura3-1 Coq9-yEGFP::Hygro Aco2-mCherry::HIS3*	This work
W303a Coq9-yeGFP Aco2-mCherry *mmm1*Δ	*MAT α, ade2-1 can1-100 his3-11,15 leu2-3,112 trp1-1 ura3-1 Coq9-yEGFP::Hygro Aco2-mCherry::HIS3 mmm1::KanMX*	This work
W303a Coq9-yeGFP Aco2-mCherry *coq11*Δ	*MAT α, ade2-1 can1-100 his3-11,15 leu2-3,112 trp1-1 ura3-1 Coq9-yEGFP::Hygro Aco2-mCherry::HIS3 coq11::LEU2*	This work
W303a Coq9-yeGFP Aco2-mCherry *mmm1*Δ *coq11*Δ	*MAT α, ade2-1 can1-100 his3-11,15 leu2-3,112 trp1-1 ura3-1 Coq9-yEGFP::Hygro Aco2-mCherry::HIS3 mmm1::KanMX coq11::LEU2*	This work
BY4741 *coq2*Δ	*MAT a his3Δ1 leu2Δ0 met15Δ0 ura3Δ0 coq2::KanMX4*	(Winzeler et al., 1999)
BY4742 *coq11*Δ	*MAT α his3Δ1 leu2Δ0 met15Δ0 ura3Δ0 coq11::LEU2*	(Bradley et al., 2020)
BY4741 *mdm10*Δ	*MAT a his3Δ1 leu2Δ0 met15Δ0 ura3Δ0 mdm10::KanMX4*	(Winzeler et al., 1999)
BY4741 *mdm10*Δ*coq11*Δ	*MAT a his3Δ1 leu2Δ0 met15Δ0 ura3Δ0 mdm10::KanMX4 coq11::LEU2*	This work
BY4741 *mdm34*Δ	*MAT a his3Δ1 leu2Δ0 met15Δ0 ura3Δ0 mdm34::KanMX4*	(Winzeler et al., 1999)
BY4741 *mdm34*Δ*coq11*Δ	*MAT a his3Δ1 leu2Δ0 met15Δ0 ura3Δ0 mdm34::KanMX4 coq11::LEU2*	This work
BY4741 + Split-MAM	*MAT α his3Δ1 leu2Δ0 lys2+/lys + met15Δ0 ura3Δ0 can1Δ::STE2pr-sp HIS5 lyp1Δ::STE3pr-LEU2; Tom20-VC-His; Sec63-VN-Kan*	(Shai et al., 2018)
BY4741 *mdm34*Δ + Split-MAM	*MAT α his3Δ1 leu2Δ0 lys2+/lys + met15Δ0 ura3Δ0 can1Δ::STE2pr-sp HIS5 lyp1Δ::STE3pr-LEU2; Tom20-VC-His; Sec63-VN-Kan; Δmdm34::Nat*	(Shai et al., 2018)

a Dr. Rodney Rothstein, Department of Human Genetics, Columbia University.

b Dr. Joan E. McEwen.

However, we were unable to generate the the *mdm12*Δ*coq11*Δ double mutant as a result of the *ERMES* mutants’ inability to retain mitochondrial DNA (Berger et al., 1997; Hobbs et al., 2001; Youngman et al., 2004; Kornmann et al., 2009; Tan et al., 2013). Despite this, the remaining mutants generated still serve as promising targets, as ERMES complex formation is abolished when any of the subunits are absent (Kornmann et al., 2009).

### Deletion of *COQ11* Improves Respiratory Growth in Select *ERMES* Mutants

**Figure 2.**
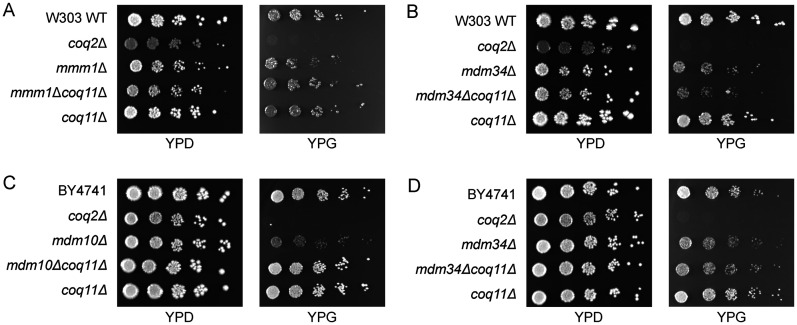
Deletion of *COQ11* improves the respiratory growth in the *mmm1*Δ and *mdm10*Δ mutants, but not in the *mdm34*Δ mutant. Spot-dilution assays were performed to assess viability of *A*, the *mmm1*Δ*coq11*Δ and *B*, *mdm34*Δ*coq11*Δ mutants on fermentable (YPD) and nonfermentable (YPG) plate medium. Yeast cells were cultured in YPG to a final *A*_600_∼1.0, harvested by centrifugation, and washed twice with sterile water. Isolated cells were resuspended and serial-diluted in sterile phosphate-buffered saline to a final *A*_600 _= 0.2, 0.04, 0.008, 0.0016, and 0.00032. Two µL of each dilution were spotted on YPD and YPG plates, and plates were incubated at 30 °C for two-to-three days. Spot dilution assays were also performed to assess viability of *C*, *mdm10*Δ*coq11*Δ and *D*, *mdm34*Δ*coq11*Δ mutants in the BY4741 genetic background. Assays were performed as described for *A* and *B*. Images are representative of at least three biological replicates.

Deletion of *COQ11* improves the respiratory growth of both the *mmm1*Δ and *mdm10*Δ mutant strains on the nonfermentable medium YPGlycerol (YPG) (Figure 2A and C). In contrast, the *mdm34*Δ*coq11*Δ mutant across two genetic backgrounds maintained the sickly growth on YPG like the *mdm34*Δ mutant (Figure 2B and D). We found it particularly interesting that the *mdm10*Δ*coq11*Δ and *mdm34*Δ*coq11*Δ mutants exhibited opposite respiratory growth phenotypes, as their respective genes encode the two mitochondrial components of ERMES that directly interact within the ERMES complex (Ellenrieder et al., 2016). However, it is not uncommon for suppressors of *ERMES* defects to rescue select *ERMES* mutants or rescue the individual deletion mutants to varying degrees (Kornmann et al., 2009; Tan et al., 2013). Additionally, it was surprising to find that deletion of *COQ11* improved the respiratory growth of the *mmm1*Δ mutant (Figure 2A). Considering Mdm12 protein stability is contingent upon stable expression of Mmm1 (Meisinger et al., 2007), and that Mmm1, Mdm12, and Mdm34 protein levels are not altered in *mdm10*Δ mutants (Ellenrieder et al., 2016), we selected the *mmm1*Δ*coq11*Δ as the most representative *ERMES*Δ*coq11*Δ mutant for further phenotypic characterization.

### Organization of the CoQ Synthome is Altered in the *mmm1*Δ*coq11*Δ Double Mutant

**Figure 3.**
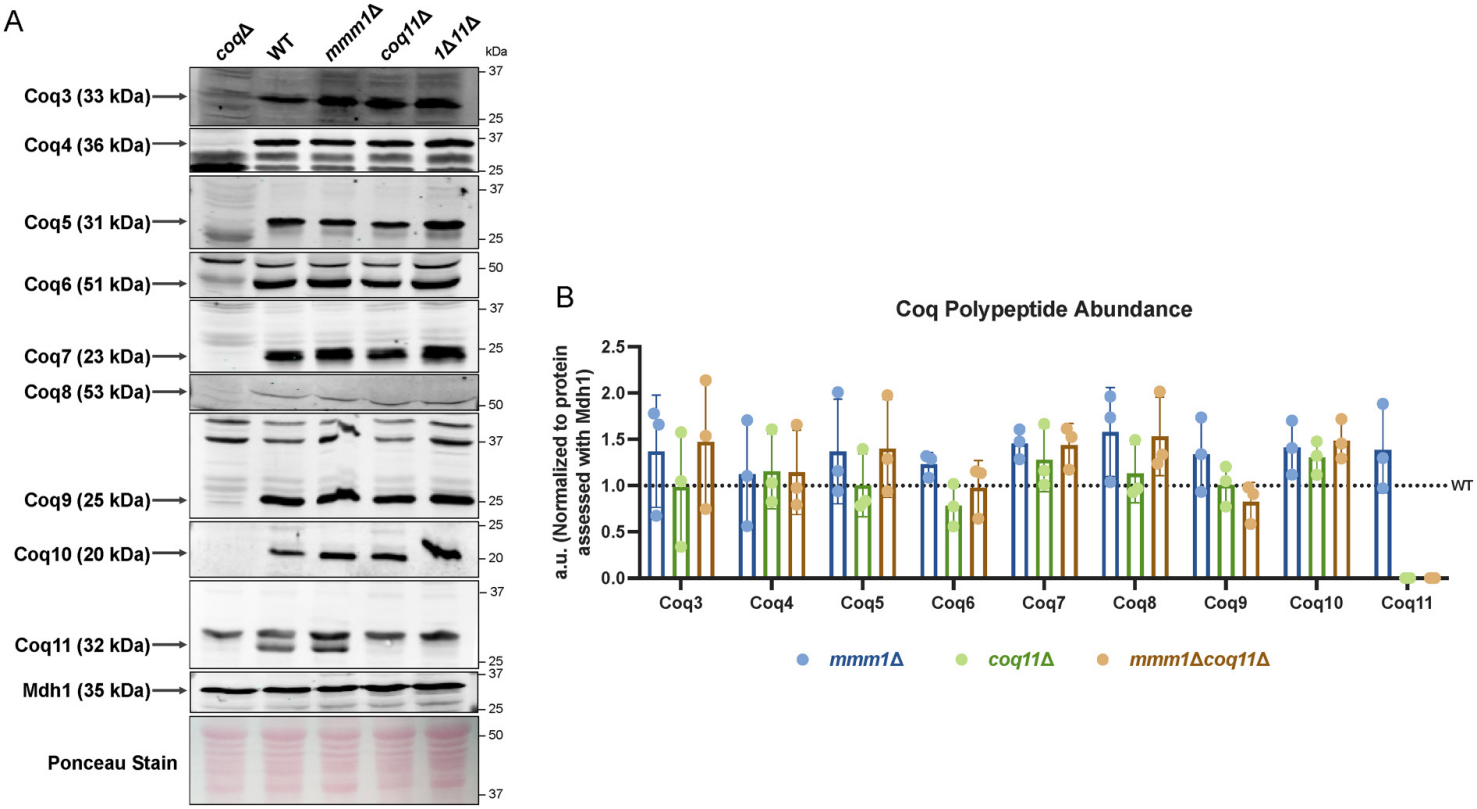
Coq polypeptide content is unaltered in the *mmm1*Δ, *coq11*Δ, and *mmm1*Δ*coq11*Δ mutants as compared to WT. *A*, Aliquots of crude mitochondria (25 µg) from wild-type (WT), *mmm1*Δ, *coq11*Δ, and *mmm1*Δ*coq11*Δ (*1*Δ*11*Δ) yeast strains were subjected to 10% or 12% Tris-glycine SDS-PAGE. Proteins stained with Ponceau stain and antisera to mitochondrial malate dehydrogenase (Mdh1) were used as loading controls. Mitochondria isolated from *coq3*Δ–*coq11*Δ (*coq*Δ) strains were used as negative controls. Black arrows indicate the location of each protein on the membrane. *B*, Band intensities corresponding to the Coq proteins were quantified by hand using ImageJ, and were normalized to Mdh1 and plotted as a percentage of WT. The data show mean ± SD of three biological replicates. A one-way analysis of variance with Dunnett's multiple comparisons showed no statistical significance.

Compared to the *coq10*Δ single mutant, which has diminished Coq polypeptide content (Allan et al., 2013; Tsui et al., 2019), the *coq10*Δ*coq11*Δ double mutant contained elevated levels of select Coq proteins (Bradley et al., 2020). Considering *ERMES* deletion mutants contain Coq polypeptide levels similar to wild-type yeast (Eisenberg-Bord et al., 2019), we were curious if the *mmm1*Δ*coq11*Δ mutant would have augmented Coq polypeptide content. Using isolated mitochondria, we performed immunoblot analyses against the identified members of the CoQ synthome, Coq3-Coq9 and Coq11, as well as Coq10. Figure 3 shows the Coq polypeptide content is unaltered in the *mmm1*Δ, *coq11*Δ, and *mmm1*Δ*coq11*Δ mutants as compared to WT. The content of the Coq1 or Coq2 polypeptides was not examined as their polypeptide content remains invariant in each of the *coq* null mutant strains (Hsieh et al., 2007; Xie et al., 2011; He et al., 2014).

**Figure 4.**
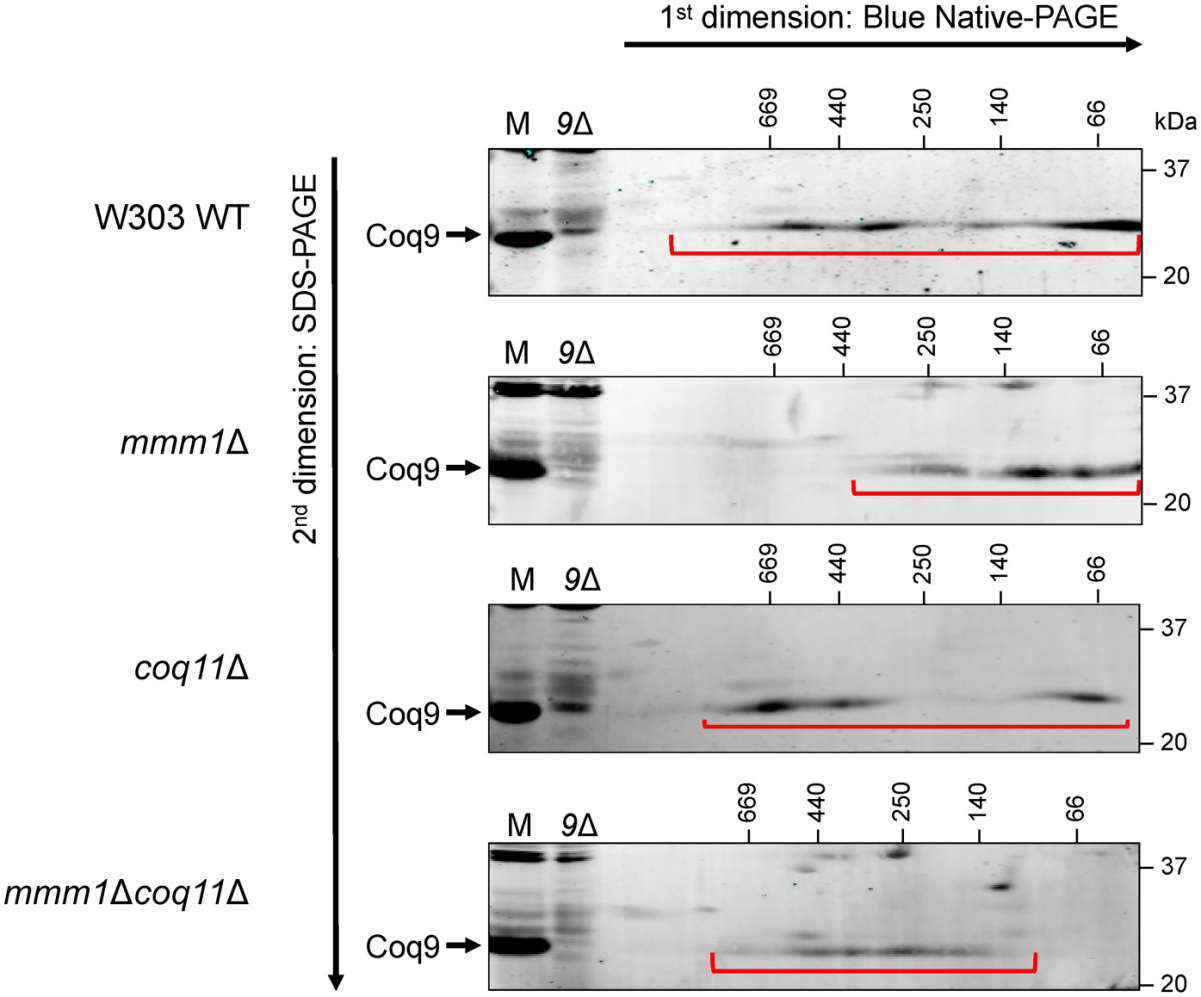
The organization of the CoQ synthome is altered in the *mmm1*Δ*coq11*Δ mutant. Digitonin-solubilized crude mitochondria from the indicated strains were subjected to two-dimensional Blue Native/SDS-PAGE (2D BN/SDS-PAGE). The CoQ synthome can be visualized in the W303 wild-type (WT) control as a heterogeneous signal between ∼66 kDa and ∼669 kDa when probed using an antibody against the Coq9 polypeptide. The signals corresponding to the CoQ synthome are indicated with the red bracket. 25 µg of intact mitochondria of the indicated strains (M) were included as a positive control, and mitochondria from *coq9*Δ yeast (*9*Δ) were included as a negative control. Data are representative of three biological replicates.

Despite the similar steady state Coq protein content, yeast lacking ERMES exhibit a dramatically destabilized CoQ synthome (Eisenberg-Bord et al., 2019). Using two-dimensional Blue Native/SDS-PAGE (2D BN/SDS-PAGE), a stable CoQ synthome can be visualized as a heterogeneous signal between ∼66 kDa and ∼669 kDa when using antisera to the Coq9 polypeptide (Hsieh et al., 2007). However, the CoQ synthome instead migrates to ∼440 kDa or less in all *ERMES* deletion mutants (Eisenberg-Bord et al., 2019), which we also observed in our analysis of the *mmm1*Δ mutant (Figure 4). Although all mutants possess similar levels of each Coq polypeptide, the signal representing the CoQ synthome in the *mmm1*Δ*coq11*Δ mutant migrates at a similar size to the wild-type control (Figure 4), but also possesses fewer smaller species (<250 kDa). This result illuminates the organizational role played by the Coq11 and Mmm1 polypeptides.

### CoQ Domain Frequency is Slightly Augmented in the *mmm1*Δ*coq11*Δ Mutant

**Figure 5.**
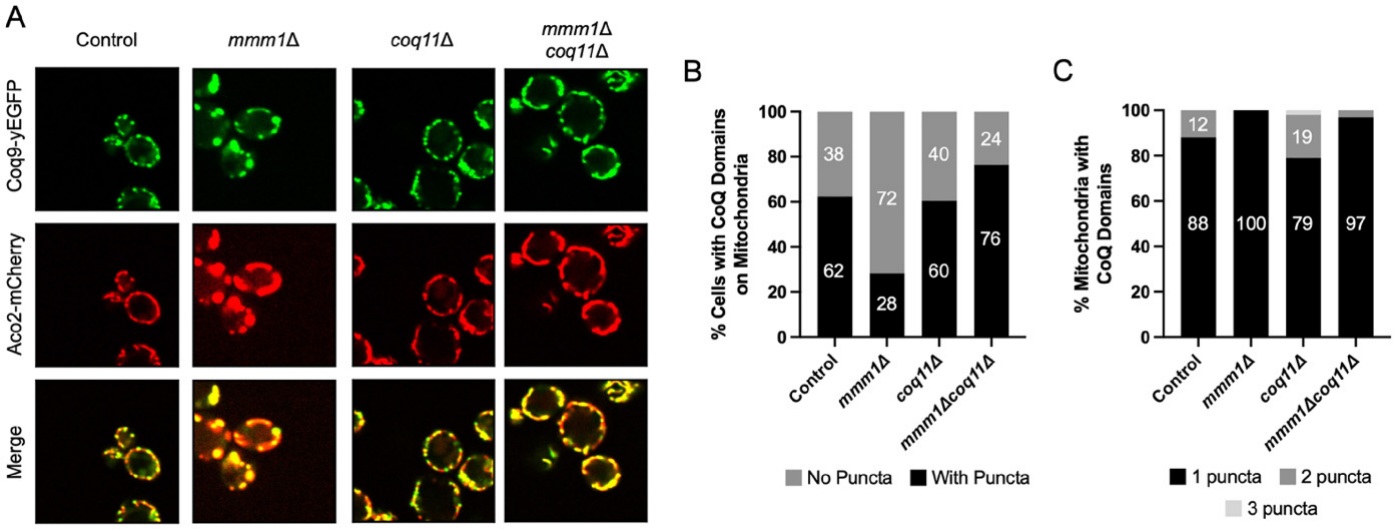
Deletion of *COQ11* slightly augments the frequency of CoQ domain formation in the *mmm1*Δ*coq11*Δ mutant. *A*, Yeast expressing yEGFP-tagged Coq9 and mCherry-tagged Aco2 as a mitochondrial marker were imaged using fluorescence microscopy. Image panels are approximately 10 µm ×10 µm. Using a neural network, Coq9-yEGFP foci were detected and plotted as *B*, percentage of cells containing CoQ domains, and *C*, percentage of total mitochondria with CoQ domains. At least 500 cells were used for these analyses.

The CoQ synthome observed using 2D BN/SDS-PAGE suggests the complex is indeed unstable, such that the migration pattern represents several smaller subcomplexes corresponding to lower molecular mass. This interpretation of these biochemical analyses is supported by the stable reconstruction of a human COQ7:COQ9 subcomplex that is stably expressed in *E. coli* (Manicki et al., 2022). The CoQ synthome in the *coq10*Δ mutant also exhibits a migration pattern representative of an unstable CoQ synthome (Tsui et al., 2019; Bradley et al., 2020). However, fluorescence microscopy experiments have demonstrated that the *coq10*Δ mutant is still able to form the complex, represented as CoQ domains marked by fluorescently tagged Coq polypeptides, but in fewer quantities than wild-type cells (Eisenberg-Bord et al., 2019; Subramanian et al., 2019). To evaluate the presence of CoQ domains representative of biosynthetic complexes, we used a fluorescence imaging-based approach using strains expressing a GFP-tagged Coq9 (Coq9-yEGFP) and an mCherry-tagged aconitase (Aco2-mCherry) as a mitochondrial marker from their endogenous loci. Tagging Coq9 and Aco2 does not affect mitochondrial function as demonstrated by growth of each tagged strain on YPG that phenocopies that of the corresponding untagged control strain (Figure S1). Our results corroborate the 2D BN/SDS-PAGE analyses in that the *mmm1*Δ*coq11*Δ double mutant has an increased number of cells that contain CoQ domains as compared to the *mmm1*Δ mutant, indicating CoQ synthome formation is repaired in the *mmm1*Δ*coq11*Δ mutant (Figure 5A and B). Upon closer examination of the mitochondria that contain CoQ domains, the *mmm1*Δ and *mmm1*Δ*coq11*Δ mutants harbor one CoQ domain per mitochondrion, whereas the wild type and *coq11*Δ mutants can possess more than one CoQ domain per mitochondrion (Figure 5C). This suggests that the number of CoQ domains may be modulated by ERMES, such that when ERMES is absent only few CoQ domains can form, even in the absence of the negative effector, Coq11.

### *coq11* Mutants Accumulate CoQ_6_-intermediates and Exhibit Inefficient CoQ_6_ Biosynthesis

**Figure 6.**
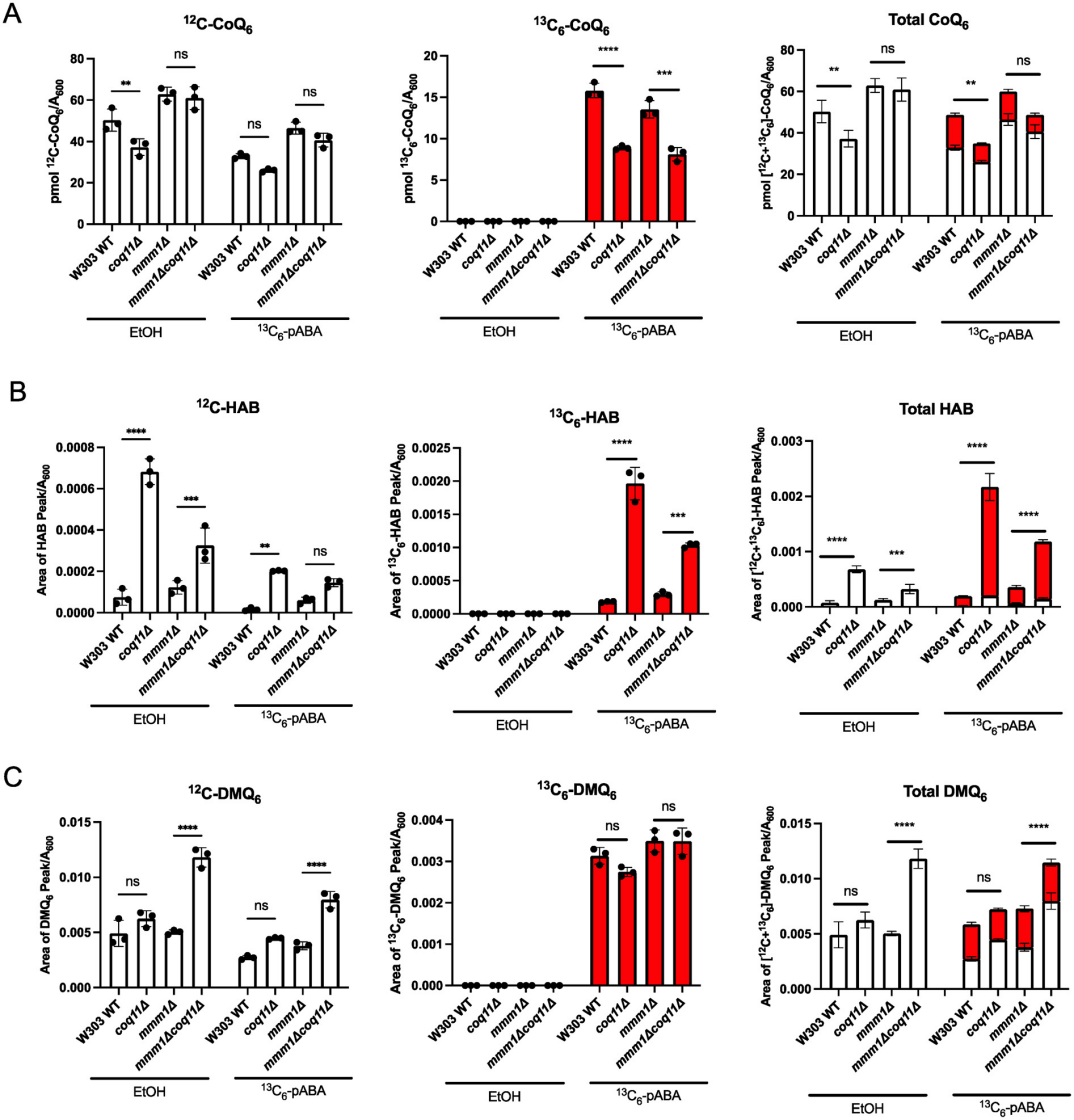
CoQ biosynthesis is inefficient in the *mmm1*Δ*coq11*Δ mutant. Triplicates of 25 mL cultures in YPG were labeled at *A*_600_∼0.6 with 8 µg/mL ^13^C_6_-para-aminobenzoic acid (^13^C_6_-pABA) or ethanol as a vehicle control. Labeled and unlabeled *A*, CoQ_6_, *B*, Hexaprenyl-aminobenzoic acid (HAB), and *C*, demethoxy-Q_6_ (DMQ_6_), were analyzed from whole cell lipid extracts after 5 h of labeling. Total content was determined using the sum of (^12^C + ^13^C_6_) of each analyte. The data depict mean ± SD of three biological replicates, and statistical significance is represented as **p < 0.01; ***p < 0.001; ****p < 0.0001; and ns, no significance. Abbreviations are defined in Figure 1. For panels *B* and *C*, 1 area unit corresponds to 4 × 10^−6^ pmol CoQ_6_.

It has been shown previously that deletion of *ERMES* results in altered *de novo* CoQ_6_ biosynthesis that is likely due to CoQ synthome destabilization (Eisenberg-Bord et al., 2019). Deletion of *COQ10* with the accompanying loss of Mdm12 due to the neighboring effects of the *HIS3* insertion cassette (Novales et al., 2024), results in a similar phenotype, which was shown to be ameliorated by subsequent deletion of *COQ11* (Bradley et al., 2020). Additionally, *de novo* CoQ biosynthesis was augmented in the *coq10*Δ*coq11*Δ mutant as compared to the *coq10*Δ single mutant (Bradley et al., 2020). These findings led us to investigate whether the altered CoQ biosynthesis in the *mmm1*Δ mutant could be remedied by deletion of *COQ11*. Yeast cultures were treated with ^13^C ring-labeled pABA and whole cell lipid extracts were analyzed using liquid chromatography with tandem mass spectrometry (LC-MS/MS). While the total CoQ_6_ content did not change between the *mmm1*Δ and *mmm1*Δ*coq11*Δ mutants, ^13^C_6_-CoQ_6_ was significantly decreased as compared to WT and the *mmm1*Δ mutant, respectively (Figure 6A). We next analyzed key intermediates, 4-amino-3-hexaprenylbenzoic acid (HAB) and demethoxy-Q_6_ (DMQ_6_), that are representative of early and late stages in the biosynthetic pathway, respectively. Consistent with previous literature (Allan et al., 2015), the *coq11*Δ mutant retained higher amounts of ^12^C-HAB and ^13^C_6_-HAB compared to the wild-type control, which the *mmm1*Δ*coq11*Δ mutant mirrors when compared to the *mmm1­*Δ mutant (Figure 6B). The *mmm1*Δ*coq11*Δ mutant possessed the highest total DMQ_6_ content, despite having *de novo* DMQ_6_ levels similar to the *mmm1*Δ mutant (Figure 6C). The elevated total DMQ_6_ content in the *mmm1*Δ*coq11*Δ mutant appears to be from the accumulation of unlabeled DMQ_6_ (Figure 6C). Taken together, the results indicate that CoQ_6_ biosynthesis is attenuated in mutants harboring the *COQ11* deletion, when compared to either WT or to the *mmm1*Δ mutant. Thus, the restoration of the CoQ synthome observed upon deletion of *COQ11* is not indicative of more efficient CoQ biosynthesis in the *mmm1*Δ*coq11*Δ mutant.

### Respiratory Growth is Unchanged in Yeast Strains Expressing Split-MAM Artificial Tether

**Figure 7.**
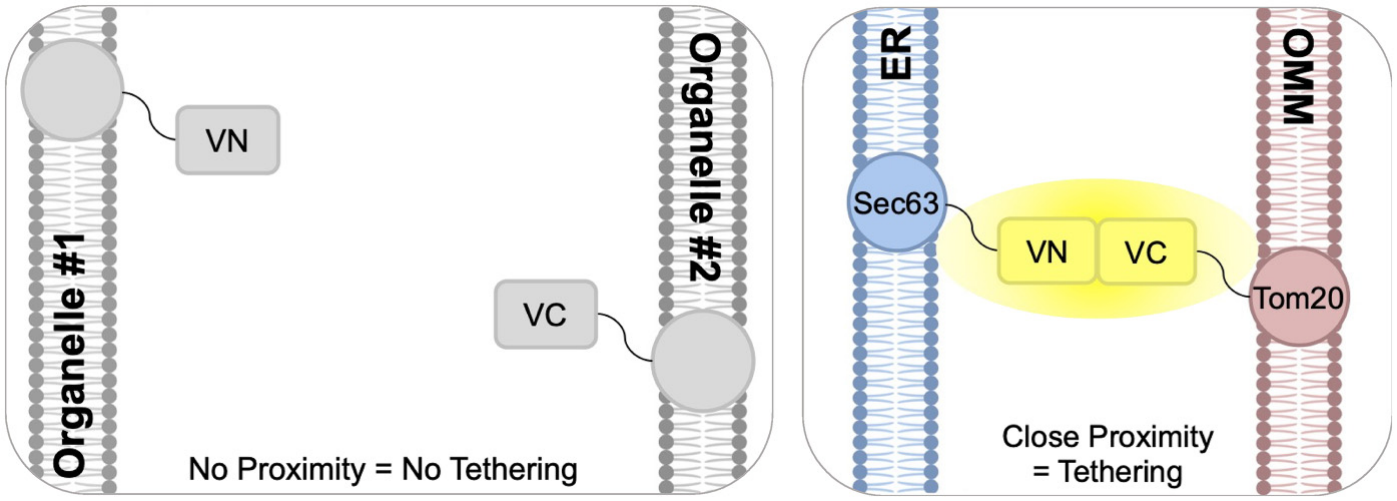
Schematic of artificial tether reporter. A Split-Venus reporter was designed having the C-terminal (VC) and N-terminal (VN) halves of the Venus fluorophore each conjugated to a membrane protein on separate organelles proposed to form a contact site. The left panel shows the general scheme; organelles that do not have the natural tendency to form a contact site will not come into proximity for the two halves to rejoin and fluoresce. The right panel illustrates the Split-MAM reporter, comprised of the outer mitochondrial membrane protein, Tom20, with the C-terminal half of Venus and the N-terminal half conjugated to the ER protein, Sec63. Formation of the tether is permitted when the organelles come into close contact, allowing the two Venus halves to recombine and fluoresce. Figure modified from (Shai et al., 2018).

**Figure 8.**
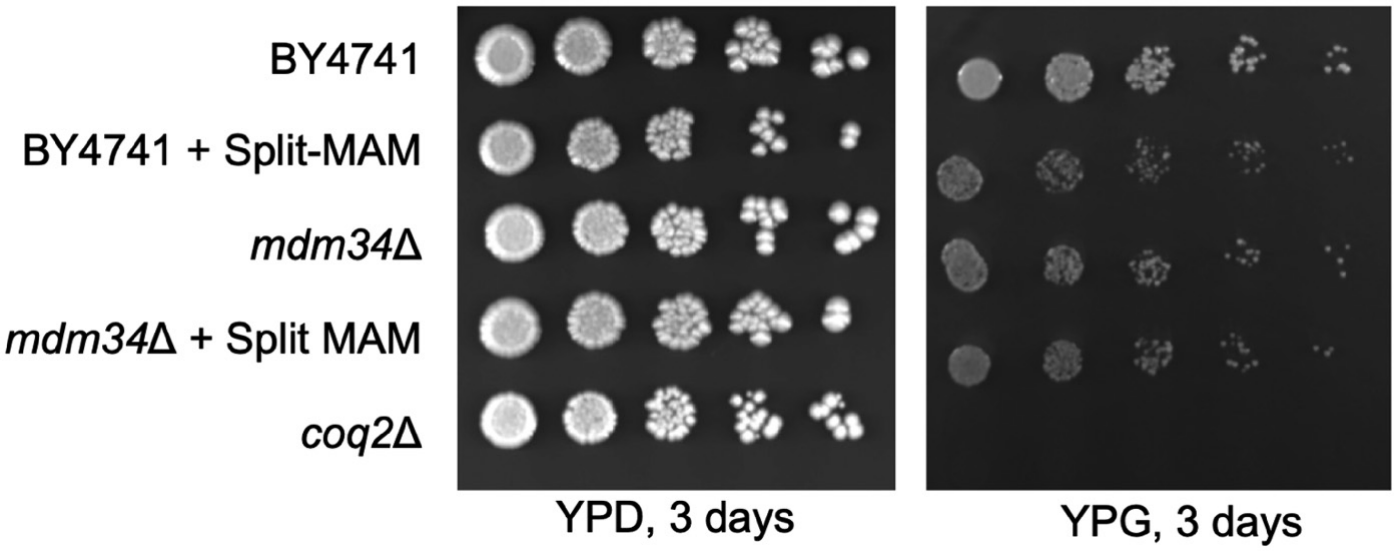
Growth on respiratory medium is unaltered in strains expressing Split-MAM compared to strains without the artificial tether. Drop dilution assay was performed as described in *Experimental Procedures*. The *coq2*Δ strain was included as a negative control, as it is unable to grow on the nonfermentable medium, YPGlycerol (YPG). Images are representative of three biological replicates.

Given the role of ERMES-mediated contact sites in coordinating CoQ biosynthesis, we sought to explore whether the introduction of an artificial tether might influence CoQ biosynthesis or content. We chose to use a variant of the Split-Venus reporter as it is designed such that the C-terminal half of the Venus fluorophore is conjugated to a protein on a candidate organelle, and the N-terminal half is conjugated to the proposed interacting organelle (Kudla and Bock, 2016; Shai et al., 2018). The ER-mitochondrial version of the Split-Venus reporter, Split-MAM (Mitochondrial Associated Membrane; illustrated in Figure 7), was shown to reinstate the ER and mitochondrial membrane apposition in the *mmm1*Δ and *mdm34*Δ mutants, demonstrating that the Split-MAM can be used to compensate for the loss of the ERMES tether (Shai et al., 2018). Using wild-type and *mdm34*Δ cells, we first evaluated the ability to grow on nonfermentable medium, with and without the expression of the Split-MAM reporter. It is of note that the *mdm34*Δ mutant in the BY4741 background does not exhibit a dramatic respiratory growth defect (Figure 8), but it was still of interest to determine if the addition of the artificial tether can bolster respiratory growth. The results of this plate viability assay show no differences in growth when the artificial tether is expressed (Figure 8).

### CoQ_6_ Content is Altered in Strains Expressing Split-MAM

**Figure 9.**
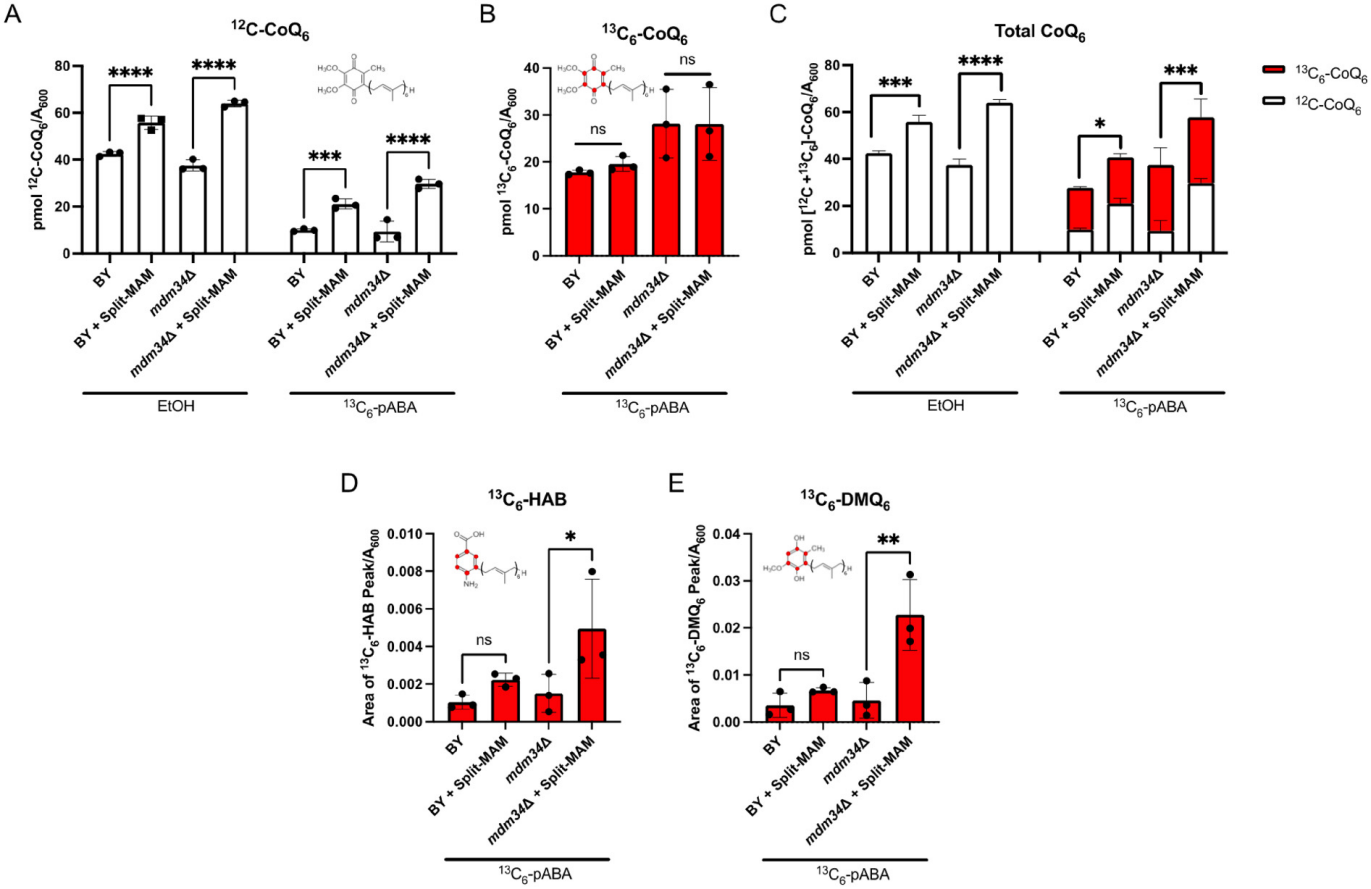
CoQ_6_ content is augmented with the expression of Split-MAM, but *de novo* CoQ_6_ content remains unchanged. Triplicate cultures of the indicated strains were grown in YPG and treated with either ^13^C_6_-pABA or ethanol vehicle control. *A*, Unlabeled ^12^C-CoQ_6_ and *B*, labeled ^13^C_6_-CoQ_6_ were measured from whole cell lipid extracts. *C*, Total CoQ_6_ was determined by taking the sum of ^12^C-CoQ_6_ and ^13^C_6_-CoQ_6_. *D* and *E*, Relative analyte levels are represented as peak area normalized to *A*_600_. Intermediate levels of ^13^C-ring labeled *D*, hexaprenyl-aminobenzoic acid (HAB) and *E*, demethoxy-Q_6_ (DMQ_6_) are elevated only in the *mdm34*Δ strain expressing Split-MAM relative to without the tether. Data show mean ± SD of three biological replicates, where each data point is the average of three technical lipid extractions. Statistical significance is represented by *p < 0.05; **p < 0.01; ***p < 0.001; ****p < 0.0001; or ns, no significance. For panels *D* and *E*, 1 area unit corresponds to 4 × 10^−6^ pmol CoQ_6_.

Given that only a small percentage of CoQ_6_ is required to observe growth on respiratory medium (Awad et al., 2018), the growth phenotype may not be representative of CoQ_6_ biosynthetic efficiency. To assess the efficiency of CoQ_6_ synthesis, we treated yeast cultures with isotopically labeled ring precursor, ^13^C_6_-pABA, or ethanol as vehicle control. It has been previously reported that *ERMES*Δ mutants, including the *mdm34*Δ mutant, contain significantly increased content of CoQ_6_ (Eisenberg-Bord et al., 2019). However, in our analyses, we observe that the content of CoQ_6_ is similar to that of the wild-type control (Figure 9A). We attribute this difference in reproducibility to genetic background, as the former study was performed in W303 yeast whereas the strains examined here are derived from the BY4741 genetic background.

Significantly elevated unlabeled CoQ_6_ content (^12^C-CoQ_6_), is observed in both wild-type and *mdm34*Δ mutant strains expressing the Split-MAM tether as compared to the respective non-expressing strains (Figure 9A). The unlabeled CoQ_6_ represents the steady state CoQ_6_ content in the ethanol control incubations, or pre-existing unlabeled CoQ_6_ in the cultures treated with the ^13^C_6_-labeled ring precursor. Interestingly, *de novo* synthesized ^13^C_6_-CoQ_6_ content remained unchanged regardless of the presence of the artificial tether (Figure 9B), indicating that the tether does not enhance the *de novo* biosynthesis. Upon evaluating total CoQ_6_ content, determined by the sum of unlabeled and ^13^C_6_-labeled CoQ_6_, we found that the accumulation of CoQ_6_ is not due to the increase in *de novo* biosynthesis, but rather the content of unlabeled CoQ_6_ (Figure 9C). In *S. cerevisiae* endogenous pABA and 4-HB are limiting for CoQ_6_ biosynthesis as addition of either aromatic precursor boosts the CoQ_6_ content (Pierrel et al., 2010). Furthermore, 4-HB is not preferentially incorporated into CoQ_6_ as compared to pABA (Pierrel et al., 2010). Thus, the increase in unlabeled CoQ_6_ in the presence of the artificial tether suggests that CoQ_6_ degradation is slowed.

The *mdm34*Δ mutant expressing Split-MAM exhibits augmented content of the early intermediate hexaprenyl-aminobenzoic acid (^13^C_6_-HAB) (Figure 9D), and the late-stage intermediate demethoxy-Q_6_ (^13^C_6_-DMQ_6_) (Figure 9E). This contrasts with the wild-type control, which does not contain elevated content of these intermediates with the additional tether (Figure 9D and E). Taken together, the results suggest that expression of the Split-MAM tether leads to the accumulation of CoQ_6_, perhaps by slowing its degradation.

## Discussion

Our results indicate that while the presence of ERMES is necessary for correct CoQ synthome assembly or stability, deletion of *COQ11* can compensate for ERMES absence*.* Deletion of *COQ11* was previously shown to rescue defects associated with *coq10*Δ yeast, including impaired respiratory growth, CoQ synthome destabilization, and inefficient CoQ biosynthesis (Bradley et al., 2020). However, the phenotypes corresponding to the destabilized CoQ synthome and inefficient CoQ biosynthesis in the *coq10*Δ mutant resulted from decreased Mdm12 protein content (Novales et al., 2024). Accordingly, we sought to determine if *COQ11* deletion could ameliorate *ERMES* deletion mutant phenotypes. In this work, we have generated several *ERMES*Δ*coq11*Δ mutants and shown that the respiratory growth of the *ERMES* mutants *mmm1*Δ and *mdm10*Δ can be significantly improved by deletion of *COQ11*.

The Coq11 polypeptide is proposed to be a negative modulator of CoQ synthome assembly, as *coq11*Δ yeast exhibit enhanced CoQ synthome formation via 2D BN/SDS-PAGE (Bradley et al., 2020) and higher intensity CoQ domains (Subramanian et al., 2019). Using the same biochemical and fluorescence-based approaches, we demonstrate that the CoQ synthome is repaired and reorganized in the *mmm1*Δ*coq11*Δ mutant and that deletion of *COQ11* slightly augments the frequency of CoQ domains relative to the *mmm1*Δ single mutant. Our characterization supports the notion that Coq11 modulates the assembly of the CoQ synthome, highlighting the interplay between ERMES, the CoQ synthome, and CoQ biosynthesis.

The SMP domain present in the Mmm1, Mdm12, and Mdm34 polypeptides subunits is a common lipid binding motif that, when aligned, can serve as a conduit for transporting lipids in and out of mitochondria (AhYoung et al., 2015; Wozny et al., 2023). Despite being unable to generate the *mdm12*Δ*coq11*Δ double mutant, we predict the *COQ11* knockout would rescue *mdm12*Δ yeast, as Mdm12 protein content relies on stable expression of *MMM1*, and vice versa (Meisinger et al., 2007; Novales et al., 2024). Intriguingly, Mmm1 protein content is depleted in *mdm34*Δ mutants, yet Mdm34 persists in *mmm1*Δ yeast (Youngman et al., 2004). This may suggest that Mdm34 is required for respiratory growth rescue mediated by deletion of *COQ11*, as the *mdm34*Δ mutant could not be rescued by *COQ11* deletion across two genetic backgrounds.

How might deletion of *COQ11* rescue of the *mmm1*Δ *ERMES* mutant? Curiously, deletion of *COQ11* impairs CoQ_6_ biosynthesis, as evidenced by elevated content of CoQ_6_ intermediates (Allan et al., 2015; Bradley et al., 2020), which we also reproduced in our analyses. Given that late-stage CoQ-intermediates are essential for CoQ synthome stability (Tran and Clarke, 2007; Subramanian et al., 2019), the deletion of *COQ11* may be rescuing CoQ synthome stability via retention of late-stage lipid intermediates, thus contributing to inefficient production of CoQ_6_. This model is feasible considering both the *mmm1*Δ and *mmm1*Δ*coq11*Δ mutants harbor Coq polypeptide content similar to WT, yet only the *mmm1*Δ*coq11*Δ double mutant, with an enhanced content of DMQ_6_, had a more stable CoQ synthome. It is also possible that deletion of *COQ11* changes the state of the CoQ synthome and/or alters the redox state of CoQ_6_ or CoQ_6_-intermediates.

Irrespective of CoQ synthome stability, strains lacking *MMM1* exhibit fewer CoQ domains per mitochondrion, suggesting that ERMES may modulate the copy number or frequency of CoQ domains. A fundamental role of membrane contact sites is to serve as recruitment sites that can help modulate inter-organelle metabolite exchange. For this reason, it would make sense that fewer contact sites due to loss of ERMES would result in fewer CoQ domains, as there would be fewer platforms to facilitate the potential distribution of CoQ.

**Figure 10.**
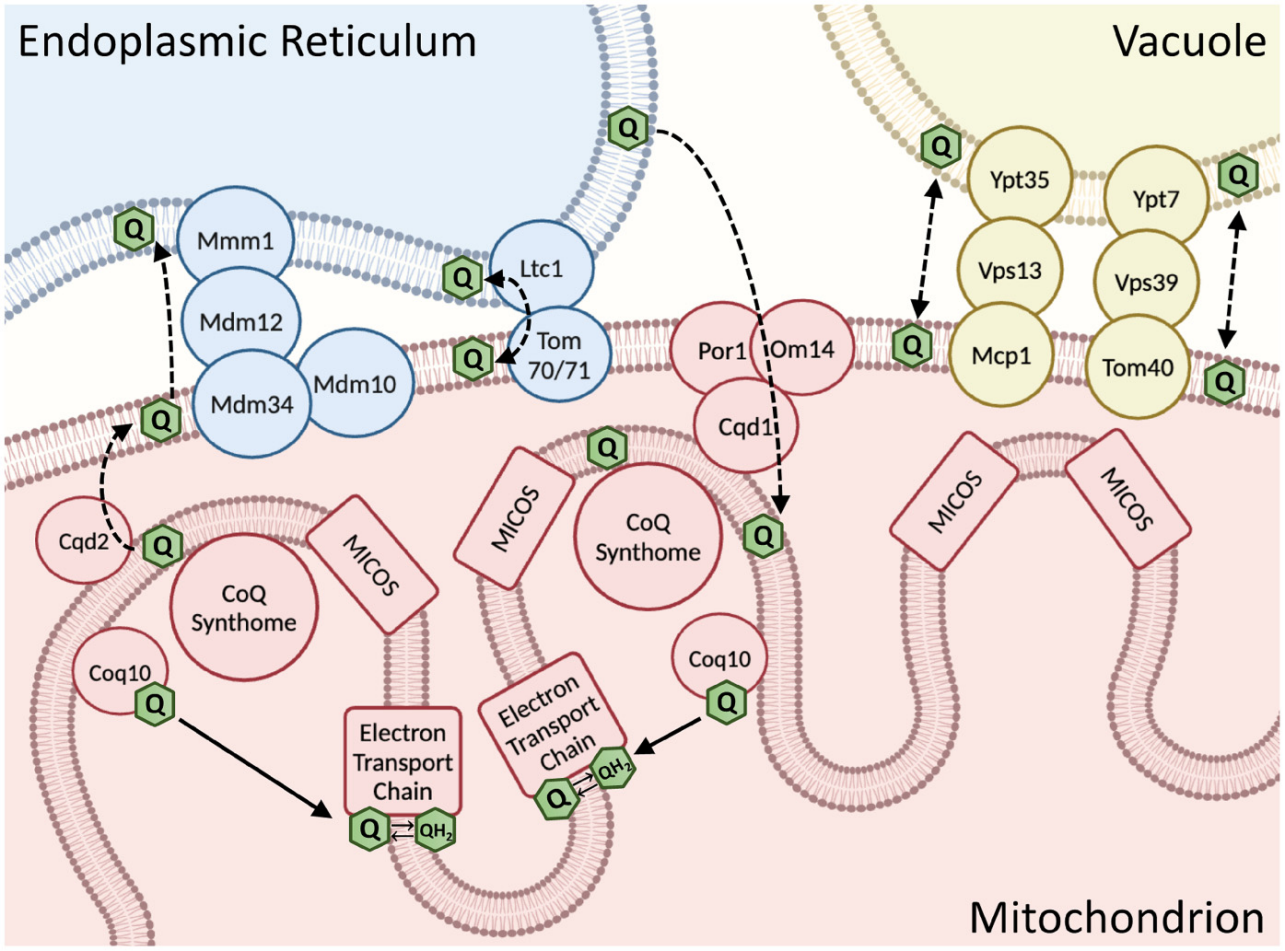
Schematic representation of mitochondrial contact sites that may be involved in CoQ trafficking. ERMES is comprised of the ER-membrane protein Mmm1, the cytosolic protein Mdm12, and the mitochondrial outer membrane proteins Mdm10 and Mdm34. It is not known how CoQ_6_ is trafficked from its site of synthesis by the CoQ synthome, which is peripherally associated with the mitochondrial inner membrane, to the outer mitochondrial membrane and to other cellular membrane destinations. Candidates for CoQ_6_ distribution are the CoQ distribution proteins 1 and 2 (Cqd1 and Cqd2) that are intermembrane space proteins, peripherally associated with the mitochondrial inner membrane. Dotted lines represent proposed routes for CoQ trafficking. Other membrane contact sites that may influence CoQ_6_ trafficking are depicted and are discussed in the text. This figure was generated using Biorender.com.

The CoQ synthome resides exclusively in the mitochondrial matrix, while ERMES tethers the outer mitochondrial membrane to the ER (Figure 10). This raises the question: how is CoQ trafficked across the mitochondrial intermembrane space (IMS)? Recently, two IMS-localized proteins have been implicated in facilitating CoQ movement between the mitochondrial and non-mitochondrial membranes (Kemmerer et al., 2021). These proteins, named CoQ distribution proteins 1 and 2 (Cqd1 and Cqd2; UNIPROT ID Q02981 and Q06567, respectively), are homologs of the Coq8 polypeptide and reciprocally regulate the distribution of CoQ in and out of the mitochondria. Specifically, deletion of *CQD1* results in excess CoQ in nonmitochondrial membranes, as determined by lipid analyses of subcellular fractions, and deletion of *CQD2* results in enhanced CoQ content within mitochondria (Kemmerer et al., 2021). Both proteins are peripherally associated to the inner mitochondrial membrane facing the IMS (Kemmerer et al., 2021), so their mode of mobilizing CoQ would still require a mechanism to transfer CoQ between the inner and outer mitochondrial membranes. Interestingly, co-deletion of *CQD1* and *CQD2* restores normal CoQ distribution, indicating redundant CoQ trafficking proteins that have yet to be identified (Kemmerer et al., 2021).

Prior to being named Cqd2, the *YLR253W* gene product was named Mcp2, (Mdm10 complementing protein 2) (Tan et al., 2013). Mdm10, a constituent of ERMES, possesses a β-barrel core structure, similar to that of VDAC and Tom40 (Flinner et al., 2013; Ellenrieder et al., 2016). Instead of binding lipids, Mdm10 functions with the sorting and assembly machinery (SAM) complex, facilitating TOM complex biogenesis and subsequent mitochondrial protein import (Becker et al., 2008). Considering this additional role of Mdm10, we found it surprising that the *COQ11* deletion robustly rescued the respiratory growth phenotype of the *mdm10*Δ mutant. Previous work has demonstrated that overexpression of Mcp2/Cqd2 suppresses the respiratory growth defect of *mdm10*Δ mutants (Tan et al., 2013). Given that overexpression of Mcp2/Cqd2 rescues *mdm10*Δ respiratory growth and modulates CoQ distribution, it is plausible that CoQ has a unique role in this suppression mechanism, which could be investigated further in our *mdm10*Δ*coq11*Δ mutant.

*MCP1* overexpression also rescues the *mdm10*Δ mutant (Tan et al., 2013). Mcp1 (UNIPROT ID Q12106), an integral outer mitochondrial membrane protein with phospholipid scramblase activity (Adlakha et al., 2022), forms a tether with Vps13 (UNIPROT ID Q07878), a lipid transport protein known to develop mutations that suppress *ERMES* mutant defects (John Peter et al., 2017; Leonzino et al., 2021). The Mcp1-Vps13 complex tethers the mitochondria and vacuole via Ypt35 (UNIPROT ID P38815), an adaptor protein with a Vps13 recognition site (Bean et al., 2018). Together, this forms a mitochondrial-vacuolar contact site redundant to the vacuole and mitochondria patch (vCLAMP) contact, comprised of Vps39, Ypt7, and Tom40 (UNIPROT ID Q07468, P32939, and P23644, respectively) (John Peter et al., 2017; Gonzalez Montoro et al., 2018). Overexpression of vCLAMP shifts the mitochondrial cristae organizing system (MICOS) from ERMES contact sites to vacuolar-mitochondrial contact sites (Tirrell et al., 2020), which is likely how *MCP1* overexpression compensates *ERMES* mutant defects.

The CoQ domains also colocalize to a redundant ER-mitochondrial contact site marked by Ltc1 (lipid transfer at contact site 1; UNIPROT ID Q08001), suggesting that the coordination of CoQ synthome assembly is not specific to ERMES, but rather any membrane contact site (Subramanian et al., 2019). Deletion of both *MDM34* and *LTC1* is synthetic lethal, indicating they likely fulfill similar roles and deletion of one can compensate for the other (Murley et al., 2015). Indeed, deletion of *LTC1* in a temperature sensitive *mmm1-1* mutant exhibits profoundly decreased frequency of CoQ domains and impaired CoQ biosynthesis (Subramanian et al., 2019). Ltc1 could serve as an auxiliary pathway for lipid transport, as *in vitro* lipid transfer assays using liposomes have demonstrated Ltc1 is capable of binding and transporting lipids (Murley et al., 2015). Clearly, there is redundancy in the function of contact sites, as the loss of ERMES enhances the number of vCLAMP contact sites and vice versa (Elbaz-Alon et al., 2014). It will be important to delineate CoQ transport mechanisms, including those mediated by the IMS-localized Cqd1 and Cqd2 proteins and auxiliary mitochondrial membrane contact sites.

Akin to *COQ11* deletion, the overexpression of *COQ8* and its human homologs rescues Coq polypeptide content, CoQ synthome assembly, and boosts CoQ synthesis (Xie et al., 2011; He et al., 2014). Given the homology with Cqd1 and Cqd2, and the ability of *CQD2/MCP2* overexpression to complement *mdm10*Δ mutants (Tan et al., 2013), it is of interest to investigate whether overexpression of *COQ8* can ameliorate *ERMES* mutant phenotypes. In an *in vitro* reconstitution of the CoQ metabolon, COQ8 is able to enhance CoQ biosynthesis in the presence of all other COQ metabolon members (Nicoll et al., 2024), and its activity is stimulated by cardiolipin (Reidenbach et al., 2018), suggesting that Coq8 may promote CoQ synthome assembly at sites enriched with cardiolipin. Cardiolipin content modulates membrane curvature (Ikon and Ryan, 2017), and may result in more extensive apposition of the inner and outer mitochondrial membranes. In this model, a soluble lipid transporter capable of establishing a direct pathway from the CoQ synthome to ERMES may not be required, thus providing a feasible mechanism of rescue mediated by *COQ8* overexpression. Overexpression of *COQ8* rescues the respiratory growth defect and impaired CoQ biosynthesis in the *coq10*Δ mutant, but does not bolster CoQ biosynthesis in either the *coq11*Δ or the *coq10*Δ*coq11*Δ mutants (Bradley et al., 2020). Considering some of the phenotypes observed in the *coq10*Δ mutant arise from *ERMES* dysfunction, we speculate that overexpression of *COQ8* may rescue *ERMES* mutant phenotypes, likely via a different mechanism than *COQ11* deletion.

To further explore if contact sites may influence CoQ biosynthesis or content, we utilized a chromosomally integrated ER-mitochondrial contact site reporter, Split-MAM, to evaluate the effects of tethers on CoQ_6_ biosynthesis. Expression of Split-MAM did not affect respiratory growth or enhance *de novo* CoQ biosynthesis, even in *mdm34*Δ yeast, with altered CoQ biosynthetic efficiency. However, accumulation of unlabeled CoQ_6_ in both the wild-type and *mdm34*Δ mutant strains expressing the Split-MAM tether suggests decreased turnover of pre-existing CoQ_6_. We posit that the physical tether may alter the distribution of CoQ_6_ by indirectly enhancing the stability of non-mitochondrial CoQ content. We speculate that the artificial tethers lack lipid transport machinery and perhaps compete with contact sites that may be needed to process the degradation of CoQ. This would indicate that while the membrane apposition between organelles is important, the presence of a functional conduit for metabolite transport between membranes is required for the maintenance of CoQ distribution or turnover.

In summary, this work identifies the deletion of *COQ11* as a novel suppressor of phenotypes associated with *ERMES* deletion mutants. We also show that expression of artificial ER-mitochondrial tethers influences the content and turnover of CoQ. Further study of how membrane contact sites regulate CoQ biosynthesis and trafficking will enable a better understanding of how interorganelle contact sites mediate mitochondrial respiratory homeostasis.

## Materials and Methods

### Yeast Strains and Growth Medium

*Saccharomyces cerevisiae* strains used in this study are listed in Table 1. Yeast strains were derived from W303 (Thomas and Rothstein, 1989) or BY4741 (Brachmann et al., 1998). Growth media included: YPG (1% yeast extract, 2% peptone, 3% glycerol), YPD (1% yeast extract, 2% peptone, 2% glucose), and YPGal (1% yeast extract, 2% peptone, 2% galactose, 0.1% dextrose). Synthetic dextrose/glycerol medium consisted of all components minus leucine. Plate medium contained 2% bacto-agar. Strains expressing the Split-MAM artificial tether were constructed by crossing yeast strains harboring the desired mutations and/or chromosomally integrated tethering constructs, followed by a series of plating on selection medium to obtain the final strains of interest (Tong and Boone, 2006; Shai et al., 2018).

The *COQ11* open reading frame was disrupted using the one-step gene disruption method (Rothstein, 1983). The donor DNA fragment was amplified by polymerase chain reaction (PCR) from a bona fide *coq11*Δ strain using the primers 5′AGTGTCTCCTCGTAATGCCATC3′ and 5′CAACCAAGAGGCATATCAGGC3′. PCR products were introduced into yeast cells using the lithium acetate method (Gietz and Woods, 2002). Yeast strains harboring fluorescent tags were generated via sporulation and tetrad dissection; these constructs were gifted by Drs. Jodi Nunnari and Kelly Subramanian from the Bay Area Institute at Altos Labs. Prior to performing experiments, the *rho* status of cells was confirmed either by maintaining growth on glycerol, or using JM6 and JM8 as *rho*0 test trains for strains that are not viable on YPG, such as *coq*Δ mutants (Santos-Ocana et al., 2002).

### Drop Dilution Plate Assays

Yeast cultures of W303 wild type, *mmm1*Δ, *coq11*Δ, *mmm1*Δ*coq11*Δ*,* BY4741 wild type, and *mdm34*Δ with and without the Split-MAM tether were grown overnight in 5 mL of YPG to ensure mutants lacking ERMES retain mitochondrial DNA. The following day, cultures were diluted to an *A*_600_∼0.25 in 15 mL of fresh YPG and expanded to a final *A*_600_∼1.0. Cells were harvested by centrifugation, washed with sterile water, and diluted in phosphate-buffered saline (PBS) to an *A*_600 _= 0.2. Aliquots of the cell suspensions (200 µL) were transferred to a 96-well plate and five-fold serial dilutions were performed four times, where for each dilution 40 µL of the cell suspension was added to a subsequent well of 160 µL of PBS. Two µL of each dilution were spotted on YPD and YPG plates, corresponding to a final *A*_600 _= 0.2, 0.04, 0.008, 0.0016, and 0.00032. All plates were incubated at 30 °C for 2–3 days.

### Isolation of Crude Mitochondria

Yeast strains were cultured overnight in 50 mL of YPG at 30 °C with shaking. Pre-cultures were back-diluted with YPG and grown for 24 h with shaking (30 °C, 250 rpm) until cell density reached an *A*_600_∼4. Cells were harvested and subsequently treated with Zymolyase-20T (MP Biomedicals) to produce spheroplasts. Spheroplasts were lysed using dounce homogenization and the resulting homogenate was subjected to centrifugation at 1,500×*g* to pellet cell debris including unlysed cells and nuclei. The supernatant was collected and subjected to centrifugation at 12,000×*g* to pellet mitochondria. The resulting mitochondrial pellet was washed and centrifuged again at 1,500×*g* to remove impurities. The final centrifugation was conducted at 12,500×*g*, and the crude mitochondrial pellet was resuspended in MES sorbitol buffer, frozen in liquid nitrogen, and stored at −80 °C until further use. All fractionation steps were completed in the presence of EDTA-free protease inhibitor cocktail tablets (Roche), phosphatase inhibitor cocktail set I (Sigma-Aldrich), phosphatase inhibitor cocktail set II (Sigma-Aldrich), and PMSF (Fisher Scientific), and all centrifugations were conducted at 4 °C. Protein concentration of crude extracts was determined by the bicinchoninic acid (BCA) assay (ThermoFisher Scientific). Due to the inability of the *coq*Δ mutant control strains to grow in YPG, crude mitochondria were isolated using the same protocol, with the culturing step completed in YPGal instead of YPG.

### Analyses of CoQ_6_ and CoQ_6_-intermediates and Stable Isotope Labeling

Yeast cultures were incubated overnight in 25 mL of YPG at 30 °C with shaking. The following day, pre-cultures were then back-diluted to an *A*_600_∼0.1 in fresh medium and further cultured to mid-log phase (*A*_600_∼0.6). To evaluate *de novo* biosynthesis, cells were treated with 8 µg/mL of ^13^C_6_-pABA for 5 h or ethanol as a vehicle control. ^13^C_6_-pABA was obtained from Sigma-Aldrich. Following treatment, cells were harvested by centrifugation and cell pellets were stored at −20 °C until use.

To prepare lipid extracts, cell pellets were resuspended in PBS, and 100 µL of the cell suspension were lysed by vortexing with glass beads in 2 mL of methanol with the addition of glass beads. The same amount of internal standard CoQ_4_ was added to each sample, and lipids were extracted with the addition of 2 mL petroleum ether twice. A standard curve comprised of known amounts of CoQ_6_ (Avanti Polar Lipids) and the CoQ_4_ internal standard was also extracted as described. Extracted lipids were dried with N_2_ and stored at −20 °C.

Lipid content was analyzed by LC-MS/MS as previously described (Tsui et al., 2019). Briefly, lipids were reconstituted in 200 µL of ethanol containing 0.5 mg/mL benzoquinone. Aliquots of each sample (20 µL) were injected into an API4000 linear MS/MS spectrometer (Applied Biosystems). The instrument's corresponding analysis software, Analyst version 1.4.2, was used for data acquisition and processing. CoQ_6_ content was determined by normalizing the corresponding peak area to a standard curve constructed with known amounts of CoQ_6_ and the CoQ_4_ internal standard. Standards of CoQ_6_ were obtained from Avanti Polar Lipids, and CoQ_4_ was obtained from Sigma-Aldrich. Relative levels of CoQ_6_-intermediates are represented as peak areas normalized to the internal standard and to the *A*_600_ of the harvested cell culture. A one-way ANOVA with Dunnett's multiple comparisons test was performed using GraphPad Prism 10.

### SDS-PAGE and Immunoblot Analysis of Steady-state Protein Expression

Crude mitochondria (25 µg) were resuspended in SDS sample buffer and separated by gel electrophoresis on 10% Tris-glycine polyacrylamide gels. Proteins were transferred to 0.45 µm PVDF membranes (Millipore) and blocked with blocking buffer (5% milk and 0.1% Tween-20 in PBS). Coq proteins and mitochondrial protein loading control Mdh1 were probed with rabbit polyclonal antibodies prepared in 0.5% bovine serum albumin at dilutions listed in Table 2.

**Table 2.** Description and Source of Antibodies.

Antibody	Working Dilution	Source
Coq3	1:200	(Poon et al., 1999)
Coq4	1:2,000	(Belogrudov et al., 2001)
Coq5	1:5,000	(Baba et al., 2004)
Coq6	1:200	(Gin et al., 2003)
Coq7	1:1,000	(Tran et al., 2006)
Coq8	Affinity purified, 1:30	(Hsieh et al., 2007)
Coq9	1:1,000	(Hsieh et al., 2007)
Coq10	Affinity purified, 1:400	(Tsui, 2019)
Coq11	1:500	(Bradley, 2020)
Mdh1	1:10,000	Lee McAlister-Henn^a^

a Dr. Lee McAlister-Henn, Department of Molecular Biophysics and Biochemistry, University of Texas Health Sciences Center.

IRDye 680LT IgG secondary antibodies (LiCOR) were used at a dilution of 1:20,000. Proteins were visualized using the LiCOR Odyssey Infrared Scanner (LiCOR). Immunoblots were quantified by hand using ImageJ software (National Institutes of Health, Bethesda, MD).

### Two-dimensional Blue Native/SDS-PAGE of High Molecular Weight Complexes

2D-BN/SDS-PAGE was performed as previously described (Schagger et al., 1994; Wittig et al., 2006). Crude mitochondria (300 µg) were solubilized for one hour on ice with 16 mg/mL digitonin (Biosynth) (4:1, digitonin:protein) in the presence of the same protease and phosphatase inhibitors from the mitochondrial isolation protocol. Solubilized protein was quantified using the BCA assay. Aliquots (80 µg) of solubilized mitochondria were separated on NativePAGE 4–16% Bis-Tris gels (Invitrogen) and cut into strips for the second-dimension separation. Gel strips were separated on 10% Tris-glycine polyacrylamide gels, followed by immunoblot analysis using an antibody against Coq9. Lyophilized protein used for the native gel high molecular weight standards were obtained from GE Healthcare (Sigma-Aldrich).

### Manual Fluorescence Microscopy

Yeast cells were grown overnight in YEPGly (2% peptone, 1% yeast extract, 3% glycerol) liquid media. Stationary phase cells were diluted in fresh medium and incubated for either 4 h or overnight. Back-dilution was done into synthetic minimal media (S; 0.67% (w/v) yeast nitrogen base (YNB) without amino acids and with ammonium sulphate, with 3% (w/v) glycerol, supplemented with required amino acids.

50 µL of cells in the stationary growth phase from each well were transferred to a glass-bottomed 384-well microscopy plate (Azenta Life Sciences) coated with Concanavalin A (ConA). Following 20 min of incubation at 25 °C, wells were washed two times with the imaging medium and then imaged. Cells were imaged using a fluorescent microscopy system (Olympus) with Hamamatsu Orca Flash 4.0 camera and a Yokogawa confocal spinning disk unit (CSUW1-T2) with a 50 μm pinhole disk and 100X oil lens (NA 1.3). Images were obtained with two channels: GFP (excitation wavelength 488 nm, emission filter set B525/50 nm) and mCherry (excitation 561 nm, emission filter set 617/73 nm). The imaging was performed by the scanR acquisition software (V3.2, Olympus). The cells from the microscopy images were segmented by scanR Analysis software (V3.2) using neural networks for recognition and measurement of their intensity.

## Supplemental Material

sj-docx-1-ctc-10.1177_25152564251316350 - Supplemental material for Mitochondrial-ER Contact Sites and Tethers Influence the Biosynthesis and Function of Coenzyme Q Supplemental material, sj-docx-1-ctc-10.1177_25152564251316350 for Mitochondrial-ER Contact Sites and Tethers Influence the Biosynthesis and Function of Coenzyme Q by Noelle Alexa Novales, Hadar Meyer, Yeynit Asraf, Maya Schuldiner and Catherine F. Clarke in Contact
